# Natural history and 12-month progression of multiple system atrophy in a Chinese cohort

**DOI:** 10.1186/s12883-026-05037-7

**Published:** 2026-06-12

**Authors:** Tao Feng, Han Wang, Jian Wang, Beisha Tang, Chun-feng Liu, Haibo Chen, Wei Mao, Qian Zhou, Olivier Rascol, Wassilios G. Meissner, Aroussi Bidani, Andreas Rieckmann, Daniel Oudin Åström, Piu Chan, Huifang Shang, Liying Cui

**Affiliations:** 1 Department of Neurology, Fengtai District, Beijing Tiantan Hospital, Capital Medical University, Beijing, China; 2 Department of Neurology, Peking Union Medical College Hospital, Chinese Academy of Medical Sciences, No. 1 Wangfujing, Shuaifuyuan Dongchengqu, Beijing, 10073 China; 3 Department of Neurology, Huashan Hospital of Fudan University, Shanghai, China; 4 Department of Neurology, Xiangya Hospital, Central South University, Changsha, Hunan China; 5 Department of Neurology, The Second Affiliated Hospital of Soochow University, Suzhou, China; 6 Department of Neurology, Beijing Hospital, Beijing, China; 7 Department of Neurology, Xuanwu Hospital of Capital Medical University, Beijing, China; 8 Lundbeck (Beijing) Pharmaceutical Consulting Co, Ltd., Beijing, China; 9 Departments of Clinical Pharmacology and Neurosciences, CRMR AMS, Clinical Investigation Center CIC‑1436, NeuroToul COEN Center, NS‑Park/FCRIN Network, University of Toulouse 3, Toulouse University Hospital, Inserm, Toulouse, France; 10 CHU Bordeaux, Service de Neurologie des Maladies Neurodégénératives, IMNc, CRMR AMS, NS-Park/FCRIN Network, Bordeaux, France; 11 University of Bordeaux, CNRS, IMN, UMR 5293, Bordeaux, F-33000 France; 12 Department of Medicine, University of Otago and New Zealand Brain Research Institute, Christchurch, New Zealand; 13 H. Lundbeck A/S, Ottiliavej 9, Valby, Copenhagen, DK-2500 Denmark; 14 Department of Neurology, West China Hospital, Sichuan University, Sichuan, China

**Keywords:** Multiple system atrophy, Characteristics, China

## Abstract

**Background:**

Understanding disease natural history is important for the development of potential treatments for people with MSA. We describe the natural progression of early MSA in a Chinese population.

**Methods:**

Observational, 12-month study conducted in 8 sites across China**.** Eligible participants were aged 40–75 years, with possible or probable MSA of the parkinsonian (MSA-P) or cerebellar (MSA-C) subtype, and anticipated survival of ≥ 3 years. Disease progression was analyzed using a linear mixed model of Total UMSARS (Part I + II) progression, including baseline, Month 6 and Month 12 data.

**Results:**

A total of 89 participants with a mean ± SD time since diagnosis of 0.4 ± 0.6 years were enrolled. Of these 52% had MSA-C and 48% participants had MSA-P. The mean ± SE [95%CI] rate of Total UMSARS progression was 1.27 ± 0.13 [1.01, 1.53] points per month. Participants showed a progression of 0.64 ± 0.06 [0.51, 0.76] points/month on UMSARS Part I and 0.62 ± 0.07 [0.47, 0.77] points/month on UMSARS Part II. Differences in the rates of UMSARS progression between patients with MSA-P and MSA-C were not statistically significant (*p* > 0.05).

**Conclusions:**

This is the first multicenter natural history study of MSA progression conducted in China. While prior studies have indicated a predominance of MSA-C in Asian populations, we found a more even split of MSA-C and MSA-P subtypes. In this early population, patients showed an average progression rate of ~ 15 Total UMSARS points/year; rates of progression were similar between the two subtypes and were in alignment with previous studies that assessed disease progression using UMSARS in Western populations.

**Trial registration:**

Clinicaltrals.gov, NCT05453058 (registered June 16, 2022).

**Supplementary Information:**

The online version contains supplementary material available at 10.1186/s12883-026-05037-7.

## Background

Multiple system atrophy (MSA) is clinically characterized by a variable combination of parkinsonian features, autonomic failure, cerebellar ataxia, and pyramidal signs [1]. Based on the predominant motor features at the time of clinical evaluation, MSA is classified as either MSA with predominant parkinsonism (MSA-P) or MSA with predominant cerebellar type (MSA-C). Although MSA-C and MSA-P share the core pathology of α-synuclein accumulation in oligodendroglial cells forming glial cytoplasmic inclusions, symptoms vary by affected regions: striatonigral degeneration causes parkinsonism in MSA-P, while olivopontocerebellar involvement leads to ataxia in MSA-C [1]. While parkinsonism is the predominant syndrome in 60–80% of Western patients [1], most Asian studies (Japan and South Korea) have, to date, reported a predominance of MSA-C [2–4].

Current treatments are symptomatic and have poor efficacy; hence an urgent unmet need remains for the treatment of MSA. Ongoing clinical disease-modification trials primarily focus on targeting α-synuclein or other mechanisms related to neuronal death, which are believed to contribute to numerous cellular dysfunctions in MSA [5]. However, a prerequisite for development of potential treatments is a better understanding of the natural history of the disease. In this respect, the available data are skewed towards Western [6–8] and, more recently, Japanese populations [4] while less is known about the progression of MSA in the Chinese population. Assuming that starting disease modifying treatments as early as possible will bring the most benefit to people with MSA (PwMSA), more precise clinical characterization of earlier disease is also needed.

The primary objective of the Talisman study was to describe early MSA disease progression in 6-month intervals over 12-months (i.e., baseline, 6-months, 12-months) in a Chinese cohort. In the context of our study, 'early' disease refers to patients who have been recently diagnosed with MSA and exhibit less severe symptoms, as measured by Unified Multiple System Atrophy Rating Scale (UMSARS) [9] Part I. A secondary objective was to describe occurrence of MSA subtypes and disease progression in each MSA subtype.

## Methods

### Study design and participants

This was a multicenter observational study conducted in 8 expert sites across China (NCT05453058). Study protocols and amendments were approved by the institutional ethics committee at each site and all participants provided written informed consent before participation. The study was executed in accordance with the declaration of Helsinki and the International Council for Harmonization (ICH) E6 4.8, principles of Good Clinical Practices (GCP).

Eligible participants were male or female aged 40–75 years, with possible or probable MSA of the parkinsonian subtype (MSA-P) or cerebellar subtype (MSA-C) [10], and an anticipated survival of ≥ 3 years. Participants must have experienced the onset of motor MSA symptoms within 5 years prior to the baseline visit, as documented in medical records, an UMSARS Part I score of ≤ 16 (omitting question 11 on sexual function), and a Montreal Cognitive Assessment (MoCA) score of ≥ 22. All participants were required to have a care partner who maintained ≥ 3 h per week contact with the patient and was able to accompany the patient to study visits.

Key exclusion criteria included evidence (clinical or on MRI) and/or history of any serious neurological disorder, other intracranial or systemic diseases or conditions resulting in a diagnosis other than MSA, and a family history (two or more blood relatives) with a history of MSA. All treatments were prescribed according to routine clinical practice and local regulations.

### Outcomes

Participants underwent a comprehensive assessment at baseline and were followed for up to 13 months with clinical assessment at 6 months and 12 months, or early discontinuation. Electronic case report forms were utilized for data collection, including demographic and clinical characteristics (including whether symptom onset started with motor or autonomic symptoms per medical records), comorbid conditions, and medication use related to MSA. To minimize recall bias, assessments were performed in order starting with patient reported outcomes (PROs), followed by clinical rating scales, and other clinical assessments. The patient’s previous or current response to levodopa was rated by the investigator on a 4-point scale developed for the purposes of the study (from ‘no effect’ to ‘clear effect’) or unknown.

Clinician rated assessments included the Chinese version of the UMSARS (Parts I–IV), the Clinical Global Impression of severity (CGI-S, 5-point scale, ranging from not impaired to extremely impaired) [11] and the Schwab and England Activities of Daily Living scale (SE-ADL) [12]. The Chinese translation of the UMSARS was produced in a stepwise process involving forward and backward translation, and linguistic validation using qualitative semi-structured cognitive debriefing interviews with 5 PwMSA and their 5 care partners. The psychometric properties of the Chinese UMSARS were found to be acceptable (results of the psychometric validation will be reported separately). Patient reported outcomes include the EuroQol 5-dimensions, 5-levels visual analogue scale (EQ-5D-5L VAS], the Patient’s Global Impression of severity (PGI-S, 5-point scale, ranging from no symptoms to very severe symptoms) and the Orthostatic Hypotension Questionnaire (OHQ) [13]. In addition, care partner quality of life was assessed using the Parkinsonism carers quality of life scale (PQoL-Carers) [14], and care partners also rated the patient’s disease severity using the observer-reported global impression of severity scale (OGI-S, 5-point scale, ranging from no impact of MSA to very severe impacts of MSA to the PwMSA).

### Statistical analysis

The planned sample size of 90 was based on the enrolment period of 9 months including feedback from the 8 hospitals, as well as the psychometric validation requirements for the Chinese version of UMSARS.

Longitudinal analyses were performed for the full analysis set (FAS), which included all participants who met inclusion criteria, had a valid baseline visit and ≥ 1 valid post-baseline visit. Analyses of Changes in UMSARS scores (Part I, Part II, and total score were performed using a linear mixed model allowing for random slopes and random intercepts to account for individual patient variability and MSA subtype, time (months), and the interaction between MSA subtype and time as fixed effects. Changes from baseline in other measures (including the following UMSARS Part I items: speech, swallowing, walking, urinary function, and falling) were examined descriptively.

A prespecified subgroup analysis examined disease progression on UMSARS by MSA subtype (MSA-P vs MSA-C). To explore any selective loss to follow-up in participants with more severe MSA, marginal structural modelling was used through which observations were weighted based on the inverse probability of not being censored.

In cases where patient records were missing, the available data were analyzed as recorded in the study. All statistical analyses were performed with R version 4.4.0.

## Results

**Fig. 1:**
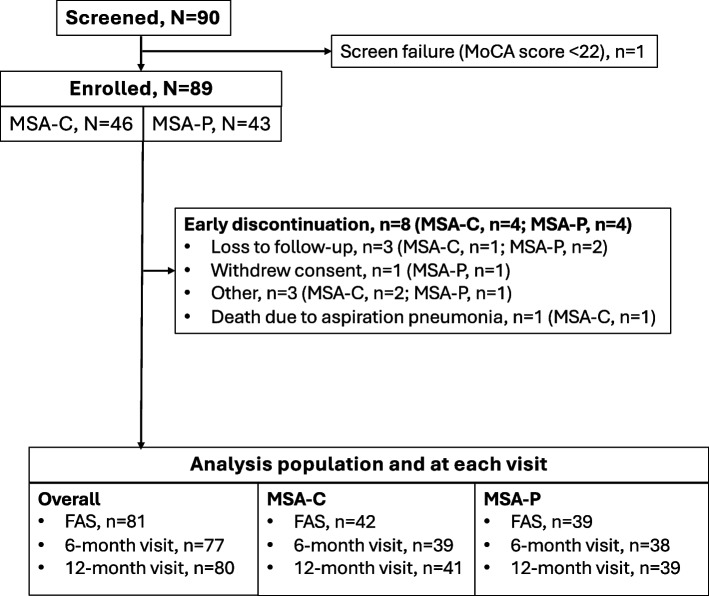
Patient disposition. Legend: FAS, full analysis set; MoCA, Montreal Cognitive Assessment; MSA-C, multiple system atrophy – cerebellar subtype; MSA-P, multiple system atrophy – parkinsonian subtype

Between June 2022 and February 2023, 90 participants were screened for eligibility, of which one had a MoCA score < 22 and was excluded from participation. Overall, 81 of 89 (91%) participants had at least one post-baseline visit (meeting criteria for inclusion in the FAS) and completed the study (Fig. 1). The main reason for early discontinuation was loss to follow up (*n* = 3) and one patient died during follow up (aspiration pneumonia at Day 379). The mean ± SD duration of follow-up was 369 ± 49 days.

Baseline characteristics are shown in Table 1. Just over half (*n* = 46 [52%]) had MSA-C and 43 [48%] participants had MSA-P. There was a predominance of males, and the mean ± SD age was 58.5 ± 6.3 years. The mean time since onset of symptoms was 2.2 years and time since MSA diagnosis was 0.4 years. About half (51%) of participants were classified as having a diagnosis of possible MSA and this was similarly distributed across both MSA subtypes. One patient changed from having a diagnosis of probable MSA-P at baseline to having a diagnosis of probable MSA-C at the 12-month follow-up. While most (*n* = 58, 72%) participants were on pharmacological treatment to improve motor symptoms, only two (3%) were rated by the investigator as having a ‘clear response’ to levodopa.

**Table 1:** Demographic and clinical characteristics at baseline

Variable	MSA-C	MSA-P	Overall
Enrolled population	*N* = 46	(*N* = 43)	(*N* = 89)
Age (years)	57.3 (6)	59.7 (6.5)	58.5 (6.3)
Sex; n (%)			
Female	12 (26%)	18 (42%)	30 (34%)
Male	34 (74%)	25 (58%)	59 (66%)
Certainty of disease diagnosis; n (%)			
Possible	19 (41%)	18 (42%)	37 (42%)
Probable	27 (59%)	25 (58%)	52 (58%)
Predominant presentation at symptom onset			
Motor	28 (61%)	30 (70%)	58 (65%)
Autonomic	10 (22%)	9 (21%)	19 (21%)
Both motor and autonomic	8 (17%)	4 (9%)	12 (13%)
Time since symptom onset (years)			
Mean (SD)	2.1 (1.1)	2.3 (0.9)	2.2 (1.0)
Median [Range]	1.9 [0.5, 5.0]	2.5 [0.5, 5.0]	2.3 [0.5, 5.0]
Time since diagnosis (years)			
Mean (SD)	0.4 (0.5)	0.5 (0.6)	0.4 (0.6)
Median [Range]	0.1 [0.0, 2.2]	0.2 [0.0, 2.4]	0.1 [0.0, 2.4]
Full analysis set (FAS)	*N* = 42	*N* = 39	*N* = 81
MSA-related treatment; n (%)			
Motor symptom medication*	26 (62%)	32 (82%)	58 (72%)
Blood pressure drugs	12 (29%)	10 (26%)	22 (27%)
Drugs for urinary symptoms	3 (7%)	2 (5%)	5 (6%)
Physiotherapy	13 (31%)	11 (28%)	24 (30%)
Levodopa; n (%) [n missing]	14 (30%) [28]	34 (79%) [5]	48 (54%) [33]
Levodopa response**; n (%)			
Clear effect	1 (6%)	1 (3%)	2 (4%)
Effect	2 (12%)	8 (22%)	10 (19%)
Little effect	4 (24%)	14 (39%)	18 (34%)
No effect	10 (59%)	12 (33%)	22 (42%)
Unknown	0	1 (3%)	1 (2%)
Missing data	*N *= 25	*N* = 3	*N* = 28
UMSARS Part I [max score 48]	12.7 (4.0)	14.1 (3.3)	13.3 (3.7)
UMSARS Part II [max score 56]	13.1 (5.2)	16.0 (5.2)	14.5 (5.4)
UMSARS I + II [max score 104]	25.8 (8.2)	30.0 (7.7)	27.8 (8.2)
UMSARS Part IV [max score 5]	1.8 (0.8)	2.0 (0.9)	1.9 (0.9)
OHSA [max score 60]	11.5 (11.6)	13.0 (8.7)	12.3 (10.3)
OHDAS [max score 40]	7.8 (8.7)	8.9 (7.5)	8.4 (8.2)
OHQ composite score [max score 100]	19.4 (18.61)	22.0 (14.80)	20.6 (16.83)

Data are mean (SD) unless otherwise stated

*ADL* Activities of daily living, *OHDAS* Orthostatic hypotension daily activity scale, *OHSA* Orthostatic hypotension symptoms assessment, *OHQ* Orthostatic Hypotension Questionnaire, *UMSARS* Unified Multiple System Atrophy Rating Scale

^*^including levodopa

^**^prior or current response

**Fig. 2:**
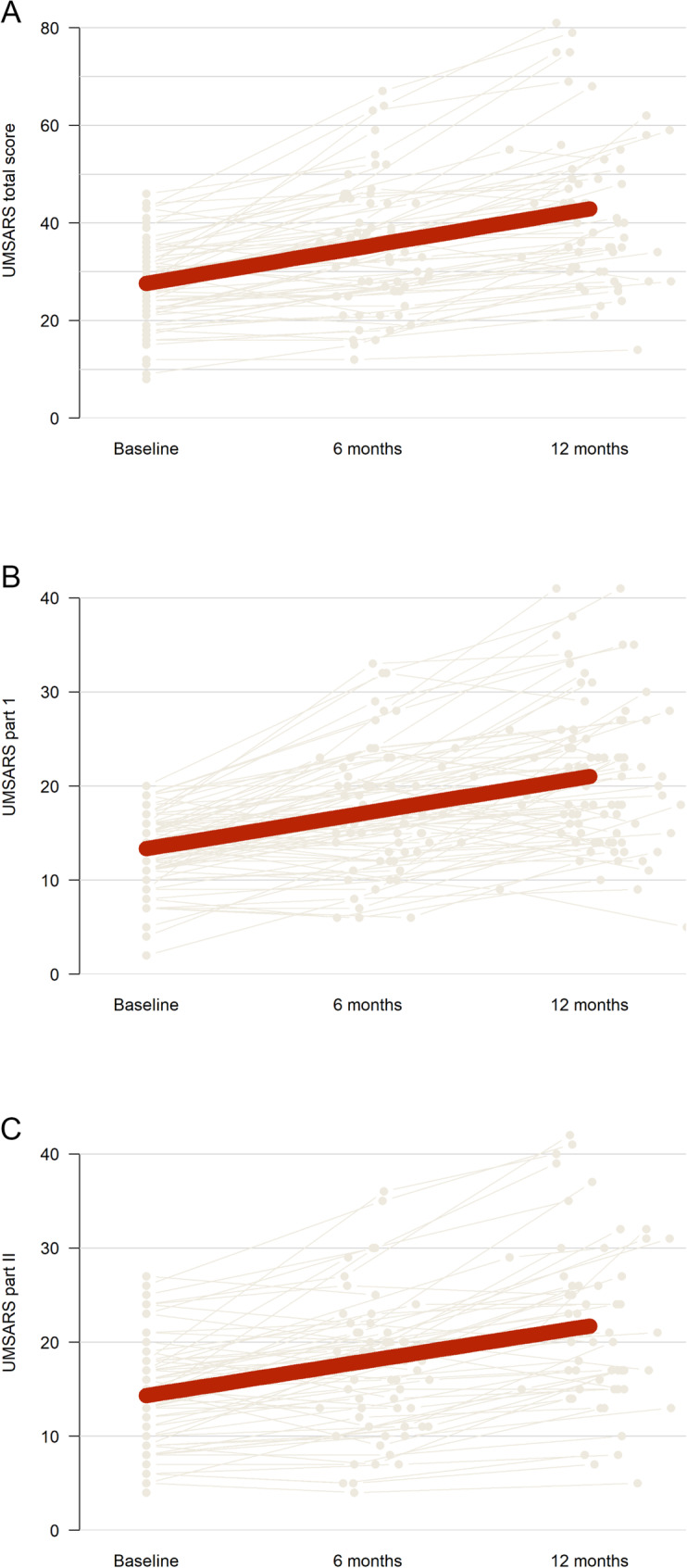
Disease progression over 12 months as assessed using the UMSARS scale (**a**) UMSARS-total score, (**b**) UMSARS Part I, (**c**) UMSARS Part II (Full analysis set). Legend: UMSARS, Unified Multiple System Atrophy Rating Scale

Participants showed a mean progression of 1.27 [95% CI 1.01 to1.53] UMSARS total score per month (Fig. 2). Rates of progression were similar for UMSARS Part I and Part II scores (Part I functional disability scores progressed by a mean of 0.64 [95% CI 0.51 to 0.76] points/month; Part II motor scores progressed by a mean of 0.62 [0.47, 0.77] points/month). Sensitivity analyses accounting for potential systematic loss to follow-up did not suggest bias.

Analysis of individual UMSARS Part I item scores showed a consistent 12-month worsening of: speech (mean ± SD change of 0.8 ± 0.88), swallowing (mean ± SD change of 0.7 ± 1.14), walking (mean ± SD change of 0.8 ± 0.87), urinary function (mean ± SD change of 0.5 ± 1.35), and falling (mean ± SD change of 0.6 ± 1.11) (Figure e2). Participants of both subtypes self-reported mild symptoms of orthostatic hypotension. For the overall cohort, composite OHQ symptoms progressed by a mean ± SD of 3.7 ± 18.64 points at Month 12.

**Fig. 3:**
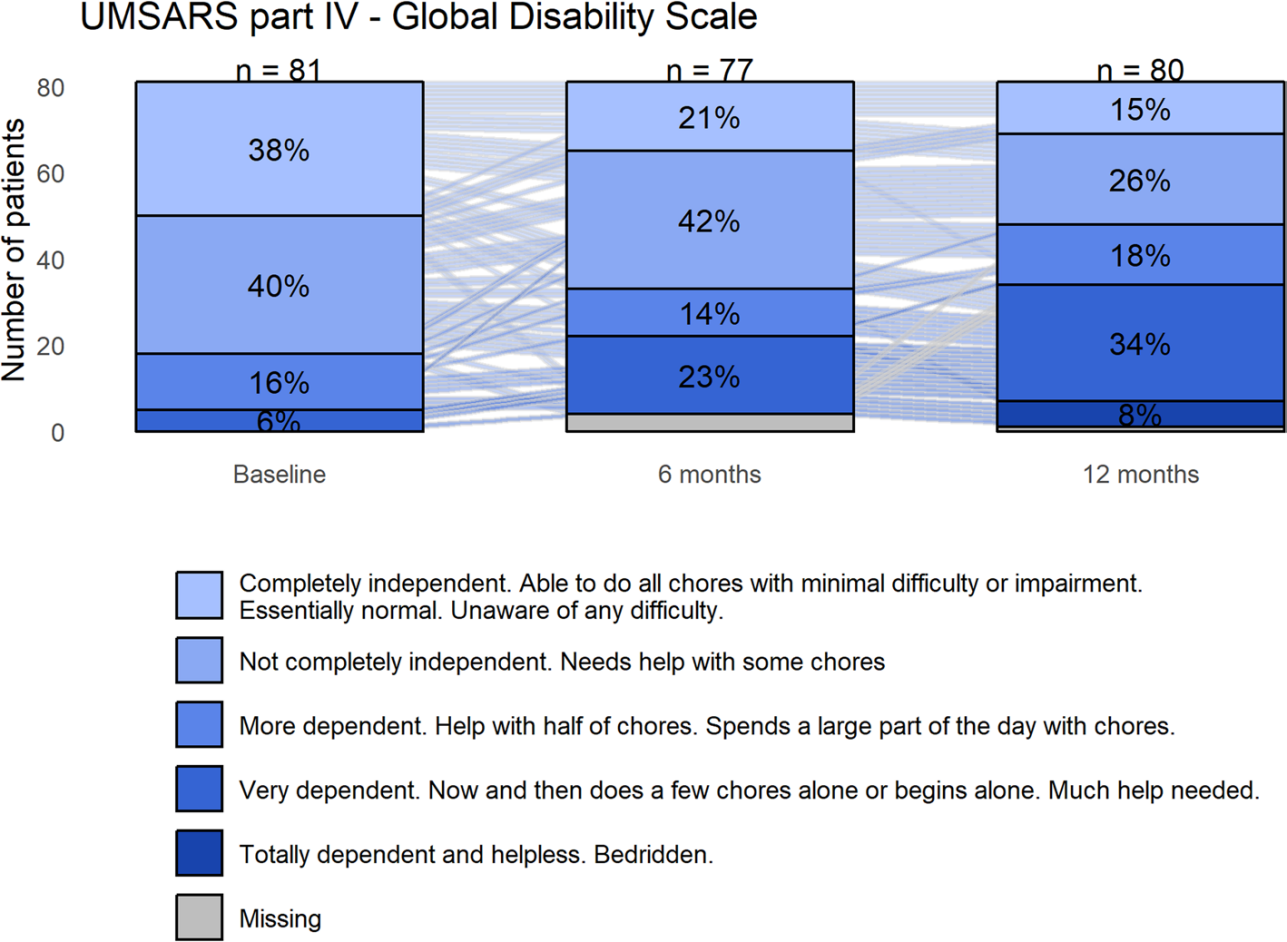
Progression of disability as assessed by UMSARS Part IV (Full analysis set). Legend: UMSARS, Unified Multiple System Atrophy Rating Scale

**Fig. 4:**
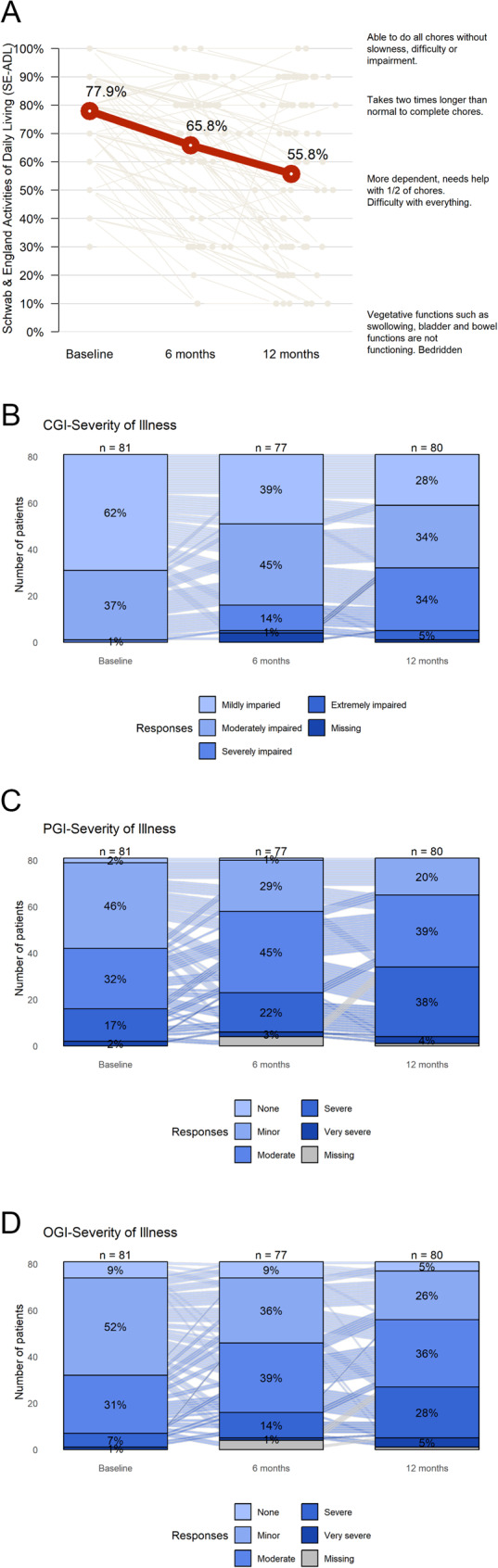
Progression of functional ability as assessed by (**a**) the Schwab and England activities of daily living scale (**b**) clinical global impression of impairment (**c**) patient global impression of symptom severity (**d**) care-partner global impression of disease impact to the patient (Full analysis set)

On average, participants showed significant worsening of disability on UMSARS Part IV, progressing from ‘not completely independent’ (mean ± SD score of 1.9 ± 0.89) at baseline to ‘more dependent’ at months 6 (score of 2.4 ± 1.07) and 12 (score of 2.92 ± 1.23) (Fig. 3). Similarly, as assessed by the Schwab and England ADL scale, participants transitioned from ‘performing chores slowly but independently’ at baseline (mean ± SD score 77.9 ± 15.55) to depending on ‘help for chores’ at month 12 (mean ± SD score 55.8 ± 28.89) (Fig. 4a). While baseline global impressions of disease severity as assessed by clinicians, PwMSA participants, and care partners showed that PwMSA often rated themselves as more severely impaired than their clinicians, all showed similarly substantial increases in the percentage of participants with severe disease (Fig. 3b-d).

Self-reported quality of life worsened over time for both PwMSA and their care-partners. PwMSA participants rated their health-related quality of life as mildly-moderately impaired (median [Q1, Q3] EQ-5D-5L VAS score of 70 [60, 85]) at baseline which progressed to moderately impaired by Month 12 (score of 60 [50, 70]). Care partner burden was rated as considerable at baseline (median [Q1, Q3] PQoL-Carers score in the overall cohort was 9.6 [5.8, 17.3]) and worsened to 13.5[5.8; 29.8] at Month 12.

Despite a similar time since onset and diagnosis, there was a non-significant tendency for faster progression on UMSARS (total score, Part I, and Part IV) and greater worsening of disability on SE-ADL, CGI, PGI, and OGI over 12 months for participants with MSA-C vs MSA-P (Figures e1-e4). UMSARS Part III autonomic examination values were similar between MSA-C and MSA-P subgroups at baseline, and no clinically relevant changes in standing/supine systolic or diastolic blood pressure were noted 6 and 12 months for either subgroup (Table e1). By Month 12, participants with MSA-C self-reported a greater worsening in quality of life compared to those with MSA-P (median [Q1, Q3] change from baseline of −10.0 [−20.0, 0.0] for MSA-C vs −5.0 [−30.0, 0.0] for MSA-P). Likewise, care-partners of participants with MSA-C self-reported a greater worsening in burden compared to those caring for participants with MSA-P (median [Q1, Q3] change from baseline of 5.8 [−1.0, 15.4] for MSA-C vs 1 [−6.7, 8.7] for MSA-P).

## Discussion

To our knowledge, this is the largest nationwide, multicenter study of MSA conducted in China and provides valuable information of clinical features of Chinese patients with MSA as assessed using both clinical rating scales and patient-reported outcomes. While some studies indicate that MSA-C may be more prevalent in South-East Asian populations, these have often been restricted to limited numbers of study sites (indicating a possible referral bias) or small sample sizes [3, 15–18]. Our multicenter study, which did not preferentially recruit one subtype over another, found a more balanced distribution. Previous Japanese studies have found that parkinsonian features may develop over time in some patients who were initially diagnosed with MSA‐C, while changes in the predominant features were not observed in MSA‐P patients [2, 15]. However, only one participant in the current cohort had a change of diagnosis during the 12-month follow-up period, which was from MSA-P to MSA-C.

Rates of clinical progression were generally in alignment with previous studies that assessed progression using UMSARS in Western populations [6–8, 19]. The rate of progression in UMSARS total score (15 UMSARS total scores per annum) appeared slightly faster in our multicenter study compared with a single center Chinese study which enrolled patients with a longer mean disease duration of 1.7 years (patients in the single center study showed a mean progression of 11.9 and 22.5 UMSARS total score at the 1- and 2-year follow-ups) [20]. As in our study, results from the North American and French registries found no significant differences in the rate of progression between MSA subtype [7, 8]. However, the consistent tendency for faster disease progression in the MSA-C subgroup in our study (across most endpoints) merits further investigation over longer periods of time. For PwMSA, the reaching of certain disease milestones often requires additional clinical attention [21], and our study characterized significant progression in the UMSARS items of speech, swallowing, walking, urinary function, and falling. For example, while most participants were rated as having easy to understand speech at baseline, the majority had to repeat statements at 12 months. Unlike the prior studies, we did not expect to observe significant mortality in this population with early MSA, and indeed only one participant died during the 12-month follow-up. Nevertheless, our findings highlight that within just 4–6 months of diagnosis, PwMSA already have relevant impairments across the autonomic and motor domains, with noticeable impairment in their global impression of disease severity.

Current pharmacological therapies for the motor features of MSA primarily target parkinsonism [22]. A retrospective single-center study of patients referred to a movement disorders clinic suggested that about half of patients with autopsy-confirmed MSA had a beneficial response to levodopa recorded in their clinical records [23], however this may reflect a predominance of MSA-P referrals to that site. In our study, while about three-quarters with MSA-P and a quarter of participants with MSA-C were receiving levodopa at baseline, only about a third were judged by the investigator as experiencing a beneficial levodopa response at baseline (increasing to half of patients in the MSA-P subgroup). A limitation of this routine practice study is that there is no consensus on the doses or duration of treatment required to conclude ‘lack of response’ and investigators may have applied various definitions according to their own clinical practice. The use of medications for autonomic dysfunction was low (< 20%) in both the MSA-C and MSA-P subgroups. However, traditional Chinese medicine is often used in the management of orthostatic hypotension, which may have led to underestimates in this study.

Of note, while both disease subtypes showed relevant impact (baseline EQ-5D-5L VAS scores ranged from 67–74 vs norms of 85–86 in the urban Chinese population aged 50–59 [24]), patients with MSA-C reported slightly worse health-related quality of life than those with MSA-P. This apparent discrepancy might be explained by factors not assessed in this study. For example, prior studies have found that, in addition to baseline disease severity and autonomic dysfunction, symptoms of depression, anxiety and cognitive function are important predictors of quality of life [18, 25]. Care partner burden was rated as considerable (well above the threshold of 62 for severe anxiety and/or depression and greater impact on quality of life [26]).

Strengths of this study lie in its prospective observational design, its naturalistic setting in several sites across China, and use of multidimensional assessment tools. However, we acknowledge several limitations. Revised criteria for MSA diagnosis [27] only became available during the study conduct, and inclusion into this study was therefore based on Gilman 2008 consensus criteria which has been criticized as not being sufficiently sensitive for the early stages. Although brain MRI markers are now required for the diagnosis of clinically established MSA, MRI findings of MSA may manifest later in the disease course, and imaging data was not routinely collected in this observational study. Distinguishing MSA from Parkinson’s disease and other atypical parkinsonian syndromes is difficult because of overlapping clinical features, often resulting in delays in establishing an MSA diagnosis [28]. Given that diagnosis is commonly delayed by about four years following motor symptom onset [8, 28], we allowed for a five-year disease course to capture this interval. The shorter diagnostic delay observed in this study may reflect the high level of expertise at the participating centers, which were selected for their experience in managing PwMSA. Of note, about one-third of participants initially presented with autonomic symptoms or a combination of autonomic and motor features, reinforcing that non-motor manifestations, such as autonomic failure, are common first manifestations of MSA [28, 29]. However, a limitation is that we did not assess autonomic features such as urogenital function in this routine practice study, which is a known prognostic factor for survival [30, 31]. Autonomic failure is increasingly recognized as an early and sometimes predominant manifestation of MSA, influencing diagnosis and prognosis and should be considered in future studies. The study was limited to 12 months, and longer time-frames are important to understand the development of clinically important milestones and mortality. We used a Chinese translation of the UMSARS, and the results from this study will be used for psychometric validation (to be reported separately). Meanwhile, a task force from the International Movement Disorder Society is currently working to update the UMSARS to improve its utility in clinical trials [32]. For example, as evidenced in this study, UMSARS items evaluating autonomic symptoms have poor ability to detect change. Finally, participants were assessed while receiving their routine therapy and treatment effects, although unlikely, cannot be ruled out.

## Conclusions

Results from this Chinese cohort with ‘early’ MSA demonstrated an average rate of clinical progression of 15 UMSARS total scores per annum, which translated into considerable worsening of disability, functioning, and overall quality of life. Although the rate of UMSARS progression was not significantly different between MSA subtypes, there was a consistent tendency for faster progression across several endpoints in participants with MSA-C compared with MSA-P.

These data provide granularity on the impact of early MSA as experienced in the Chinese population and support the feasibility of identifying and following patients who are still relatively early in their disease course, and who might be good candidates for clinical trials testing the various disease modifying interventions currently under investigation [22]. Reliable natural history study data such as presented here advance the understanding of MSA and will help facilitate proper interpretation of clinical data. Study results also highlight the rapid progression of the disease underscoring the need for earlier diagnosis and specialized disease management.

## Supplementary Information

Additional file 1: Figure e1. Disease progression over 12 months (MSA-C and MSA-P) as assessed using the UMSARS scale (a) UMSARS-total score, (b) UMSARS Part I, (c) UMSARS Part II (Full analysis set). Figure e2. Progression of UMSARS Part I items over 12 months (a) speech, (b) swallowing, (c) walking, (d) urinary function, (e) falling (Full analysis set). Figure e3. Change in UPDRS Part IV disability scores across visits for the (a) MSA-C subgroup (b) MSA-P subgroup (Full analysis set). Figure e4. Change in global impressions of severity by MSA-subtype (Full analysis set). Table e1. Changes in UMSARS Part III blood pressure monitoring.

## Data Availability

The datasets used and/or analyzed during the current study are available from the corresponding author on reasonable request.

## Abbreviations

FAS: Full analysis set
MoCA: Montreal Cognitive Assessment
MSA: Multiple system atrophy
MSA-C: Multiple system atrophy cerebellar subtype
MSA-P: Multiple system atrophy parkinsonian subtype
PwMSA: People with multiple system atrophy
UMSARS: Unified multiple system atrophy rating scale
