# Supplementary material for: Natural history and 12-month progression of multiple system atrophy in a Chinese cohort
Source: BMC Neurol. 2026 Jun 12;26:487. doi: 10.1186/s12883-026-05037-7 (PMC13425784; doi:10.1186/s12883-026-05037-7)
Supplement: Supplementary file 1 — Additional file 1: Figure e1. Disease progression over 12 months (MSA-C and MSA-P) as assessed using the UMSARS scale (a) UMSARS-total score, (b) UMSARS Part I, (c) UMSARS Part II (Full analysis set). Figure e2. Progression of UMSARS Part I items over 12 months (a) speech, (b) swallowing, (c) walking, (d) urinary function, (e) falling (Full analysis set). Figure e3. Change in UPDRS Part IV disability scores across visits for the (a) MSA-C subgroup (b) MSA-P subgroup (Full analysis set). Figure e4. Change in global impressions of severity by MSA-subtype (Full analysis set). Table e1. Changes in UMSARS Part III blood pressure monitoring. [file 12883_2026_5037_MOESM1_ESM.pdf]

Supplement

Figure e1. Disease progression over 12 months (MSA-C and MSA-P) as assessed using the UMSARS scale (a) UMSARS-total score, (b) UMSARS Part I, (c) UMSARS Part II (Full analysis set)

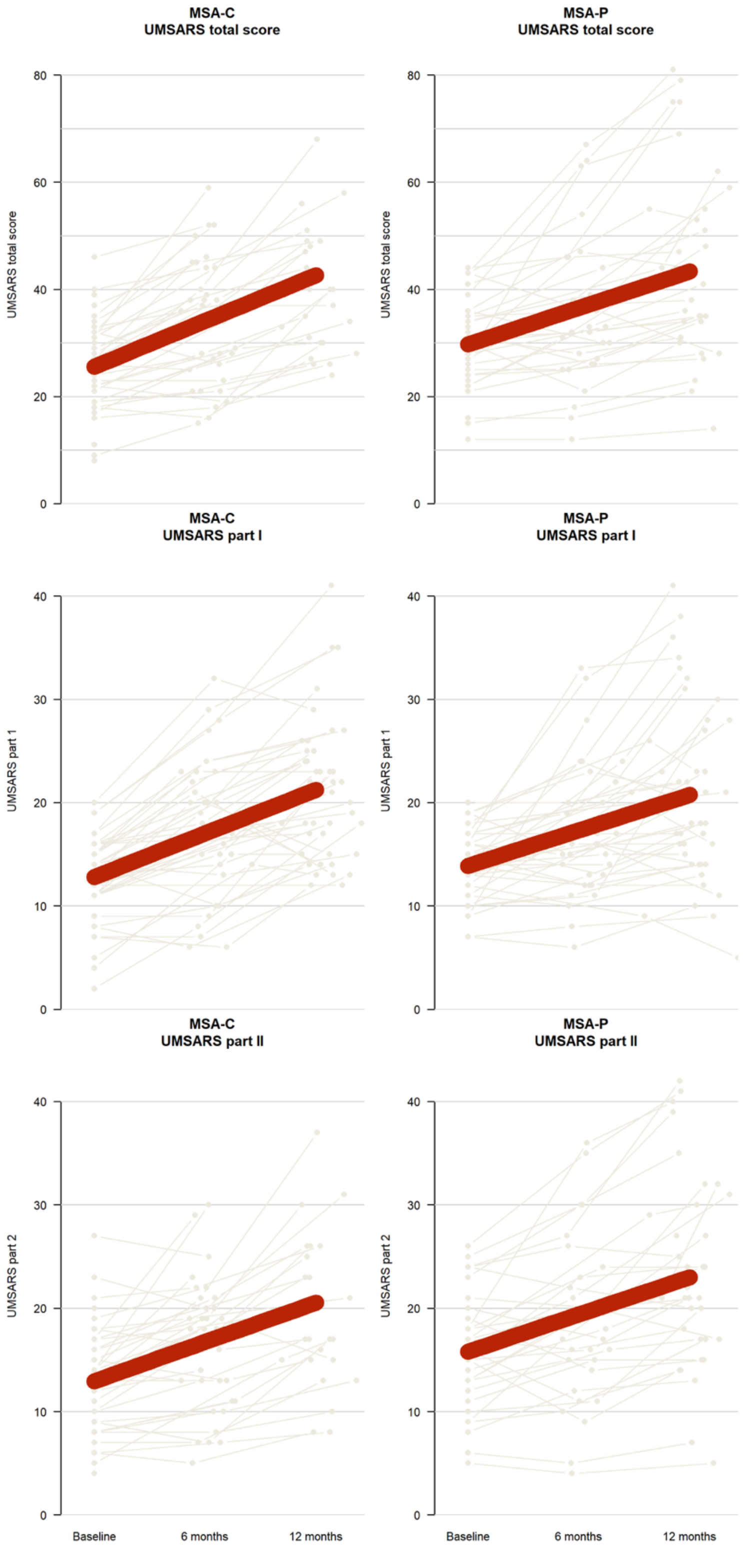

Figure e2. Progression of UMSARS Part I items over 12 months (a) speech, (b) swallowing, (c) walking, (d) urinary function, (e) falling (Full analysis set)

(a)

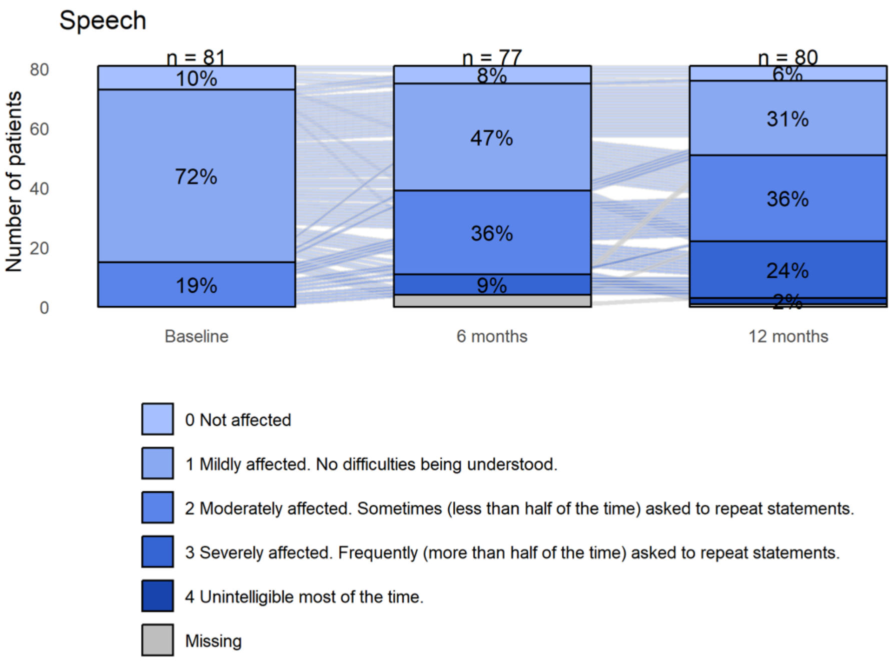

(b)

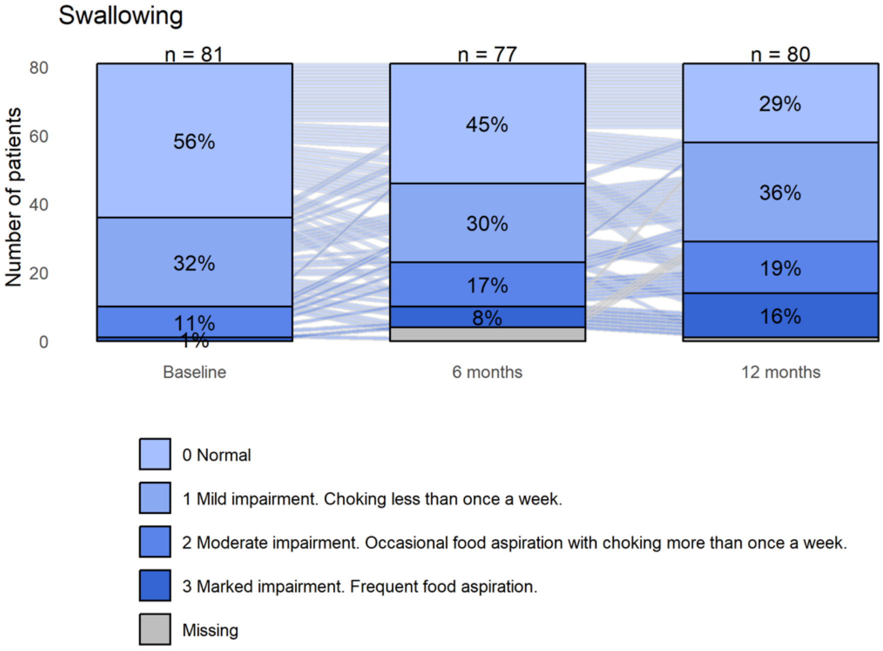

(c)

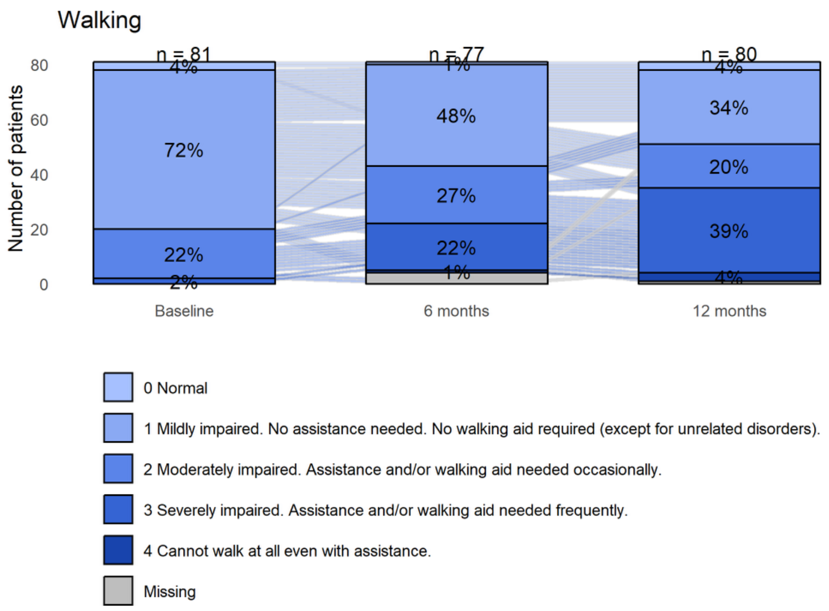
Walking
Number of patients
80
60
40
20
0
n = 81
n = 77
n = 80
4%
72%
22%
2%
1%
48%
27%
22%
1%
4%
34%
20%
39%
4%
Baseline
6 months
12 months
0 Normal
1 Mildly impaired. No assistance needed. No walking aid required (except for unrelated disorders).
2 Moderately impaired. Assistance and/or walking aid needed occasionally.
3 Severely impaired. Assistance and/or walking aid needed frequently.
4 Cannot walk at all even with assistance.
Missing

(d)

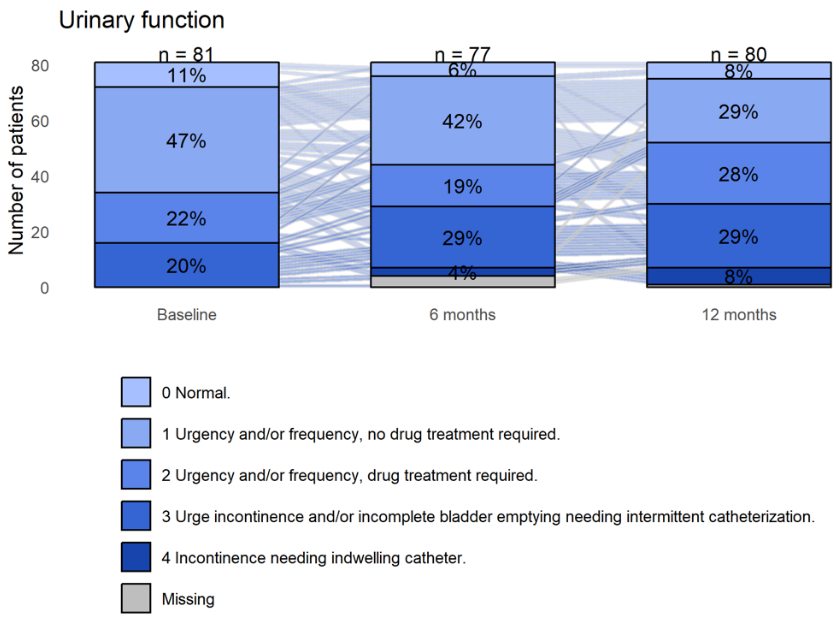
Urinary function
Number of patients
80
60
40
20
0
n = 81
n = 77
n = 80
11%
47%
22%
20%
6%
42%
19%
29%
4%
8%
29%
28%
29%
8%
Baseline
6 months
12 months
0 Normal.
1 Urgency and/or frequency, no drug treatment required.
2 Urgency and/or frequency, drug treatment required.
3 Urge incontinence and/or incomplete bladder emptying needing intermittent catheterization.
4 Incontinence needing indwelling catheter.
Missing

(e)

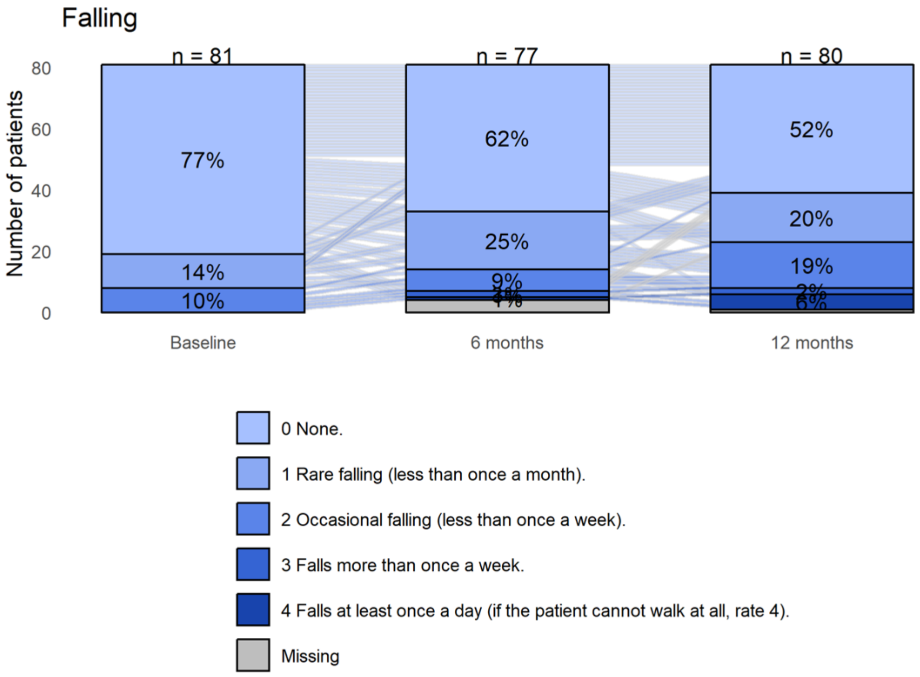
Falling
n = 81
n = 77
n = 80
Number of patients
80
60
40
20
0
77%
14%
10%
62%
25%
9%
52%
20%
19%
Baseline
6 months
12 months
0 None.
1 Rare falling (less than once a month).
2 Occasional falling (less than once a week).
3 Falls more than once a week.
4 Falls at least once a day (if the patient cannot walk at all, rate 4).
Missing

Figure e3. Change in UPDRS Part IV disability scores across visits for the (a) MSA-C subgroup (b) MSA-P subgroup (Full analysis set)

(a)

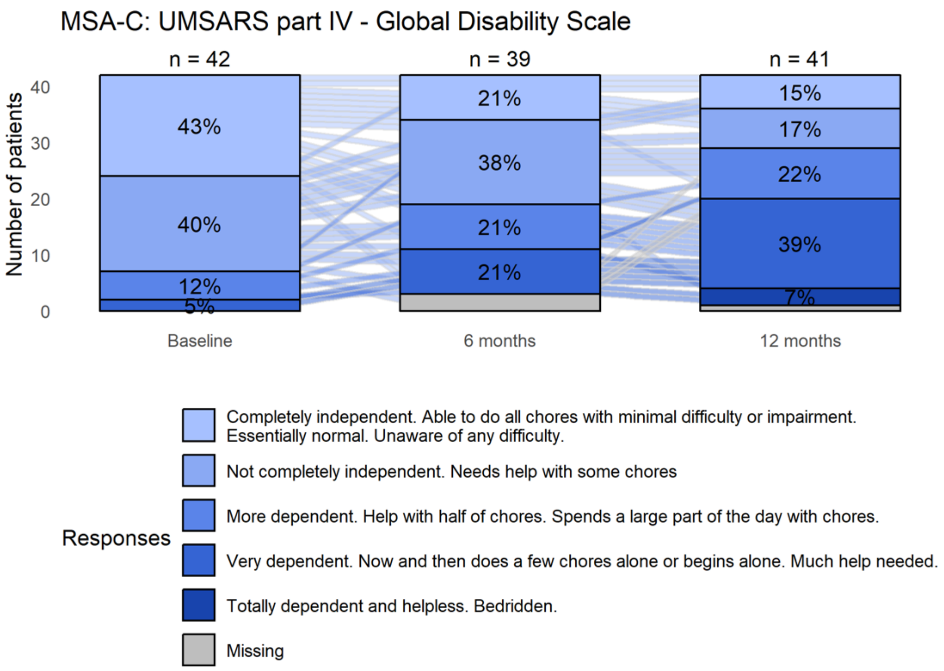

(b)

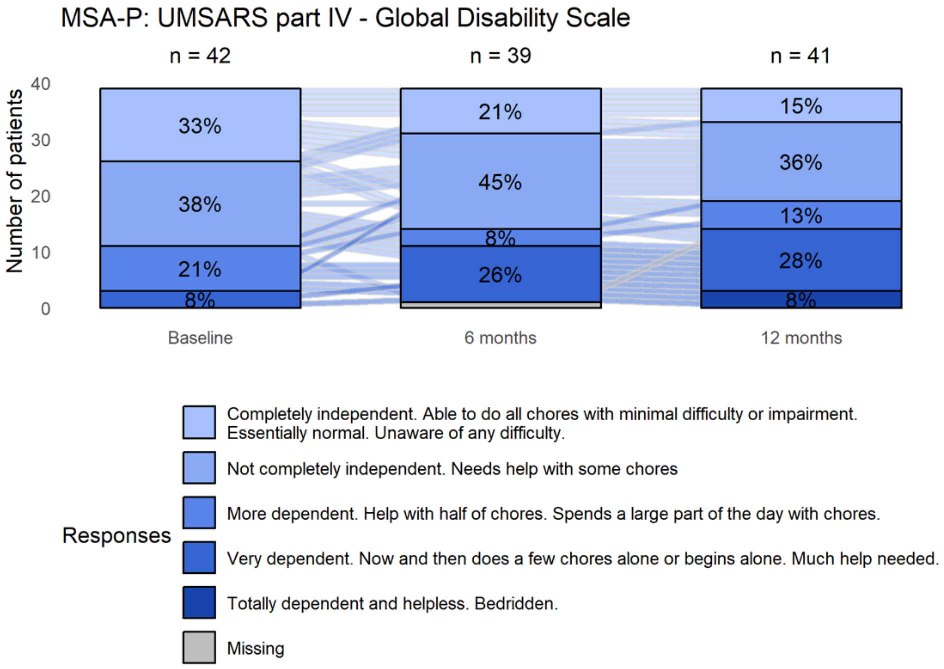

Figure e4. Change in global impressions of severity by MSA-subtype (Full analysis set)

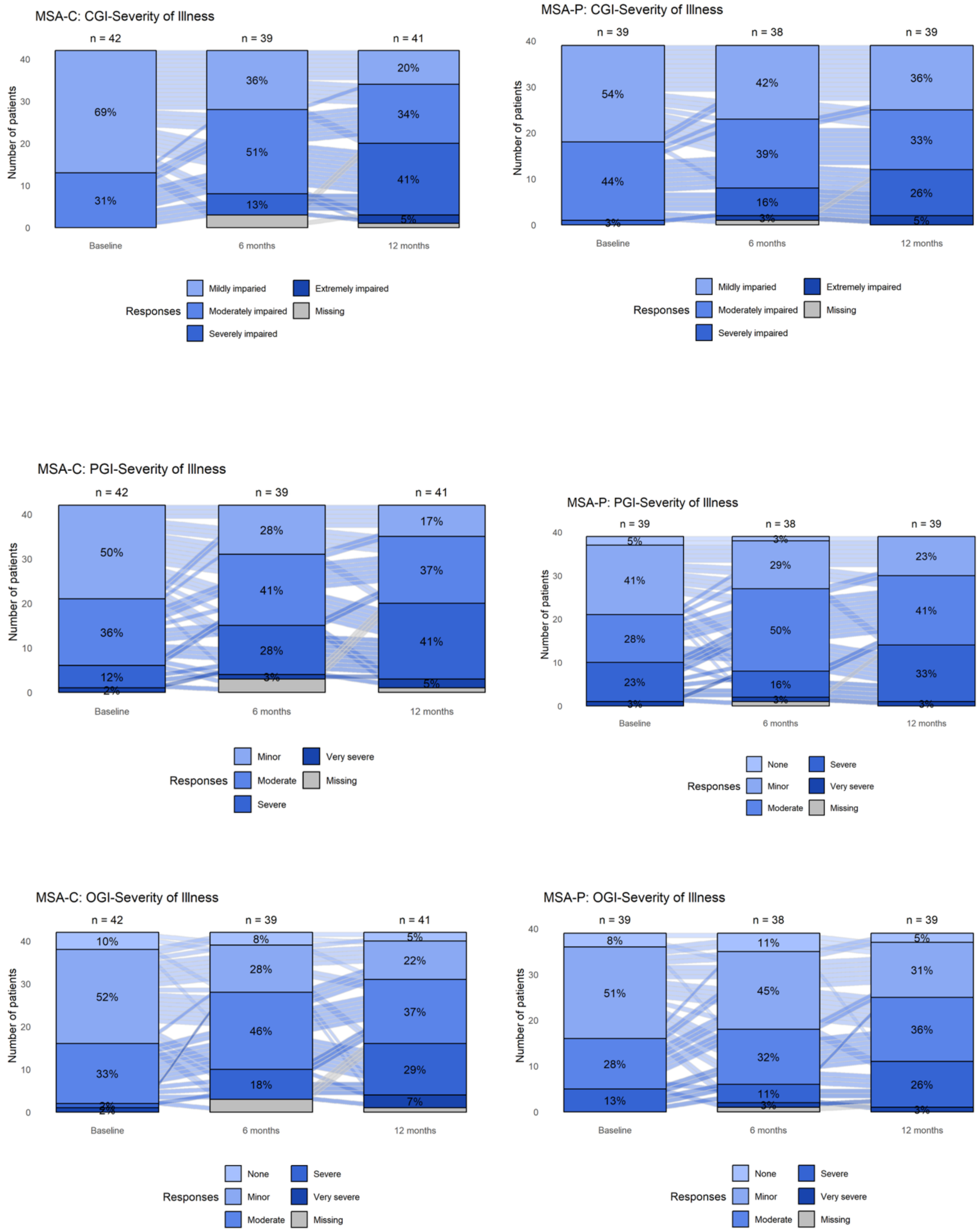

Table e1. Changes in UMSARS Part III blood pressure monitoring

	Baseline	Month 6	Month 12
UMSA1C-Systolic Blood Pressure: Supine	129.4 ± 18.63	130.1 ± 17.5	132.1 ± 19.3
UMSA1C-Systolic Blood Pressure: Standing	110.3 ± 20.9	111.1 ± 18.6	110.6 ± 20.7
UMSA1C-Diastolic Blood Pressure: Supine	84.4 ±11.8	84.0 ± 12.2	85.4 ± 12.4
UMSA1C-Diastolic Blood Pressure Standing	77.4 ± 12.6	76.6 ± 11.8	76.8 ± 12.4
UMSA1C-Heart Rate: Supine	76.0 ±11.4	77.3 ± 11.5	80.2 ± 9.9
UMSA1C-Heart Rate: Standing	83.5 ± 10.1	84.0 ± 11.6	87.2 ± 11.3

Data are mean ± SD
